# High cognitive reserve attenuates the risk of dementia associated with cardiometabolic diseases

**DOI:** 10.1186/s13195-024-01528-2

**Published:** 2024-07-19

**Authors:** Abigail Dove, Wenzhe Yang, Serhiy Dekhtyar, Jie Guo, Jiao Wang, Anna Marseglia, Davide Liborio Vetrano, Rachel A. Whitmer, Weili Xu

**Affiliations:** 1 Aging Research Center, Department of Neurobiology, Care Sciences and Society, Karolinska Institutet, Stockholm, Sweden; 2 Department of Epidemiology and Biostatistics, School of Public Health, Tianjin Medical University, Tianjin, China; 3 Department of Nutrition and Health, China Agricultural University, Beijing, China; 4 Department of Epidemiology, College of Preventive Medicine, Army Medical University, Chongqing, China; 5 Division of Clinical Geriatrics, Center for Alzheimer’s Research, Department of Neurobiology, Care Sciences and Society, Karolinska Institutet, Stockholm, Sweden; 6 Stockholm Gerontology Research Center, Stockholm, Sweden; 7 Department of Public Health Sciences and Neurology, University of California Davis, Davis, CA USA

**Keywords:** Cardiometabolic disease, Dementia, Cognitive reserve, Brain magnetic resonance imaging, Population-based follow-up study, UK Biobank

## Abstract

**Background:**

Cardiometabolic diseases (CMDs) including type 2 diabetes, heart disease, and stroke have been linked to a higher risk of dementia. We examined whether high levels of cognitive reserve (CR) can attenuate the increased dementia risk and brain pathologies associated with CMDs.

**Methods:**

Within the UK Biobank, 216,178 dementia-free participants aged ≥ 60 were followed for up to 15 years. Baseline CMDs and incident dementia were ascertained from medical records, medication use, and medical history. Latent class analysis was used to generate an indicator of CR (low, moderate, and high) based on education, occupational attainment, confiding in others, social contact, leisure activities, and television watching time. A subsample (*n* = 13,663) underwent brain MRI scans during follow-up. Volumes of total gray matter (GMV), hippocampus (HV), and white matter hyperintensities (WMHV) were ascertained, as well as mean diffusivity (MD) and fractional anisotropy (FA) in white matter tracts.

**Results:**

At baseline, 43,402 (20.1%) participants had at least one CMD. Over a mean follow-up of 11.7 years, 6,600 (3.1%) developed dementia. The presence of CMDs was associated with 57% increased risk of dementia (HR 1.57 [95% CI 1.48, 1.67]). In joint effect analysis, the HRs of dementia for people with CMDs and moderate-to-high CR and low CR were 1.78 [1.66, 1.91] and 2.13 [1.97, 2.30]), respectively (reference: CMD-free, moderate-to-high CR). Dementia risk was 17% lower (HR 0.83 [0.77, 0.91], *p* < 0.001) among people with CMDs and moderate-to-high compared to low CR. On brain MRI, CMDs were associated with smaller GMV (β -0.18 [-0.22, -0.13]) and HV (β -0.13 [-0.18, -0.08]) as well as significantly larger WMHV (β 0.06 [0.02, 0.11]) and MD (β 0.08 [0.02, 0.13]). People with CMDs and moderate-to-high compared to low CR had significantly larger GMV and HV, but no differences in WMHV, MD, or FA.

**Conclusions:**

Among people with CMDs, having a higher level of CR was associated with lower dementia risk and larger gray matter and hippocampal volumes. The results highlight a mentally and socially active life as a modifiable factor that may support cognitive and brain health among people with CMDs.

**Supplementary Information:**

The online version contains supplementary material available at 10.1186/s13195-024-01528-2.

## Background

An estimated 50 million people worldwide are currently living with dementia, and this number is projected to triple to 150 million by 2050 as the global population ages [1, 2]. Cardiometabolic diseases (CMDs) – a cluster of related diseases including type 2 diabetes (T2D), heart disease, and stroke [3] – are important risk factors for dementia [4]. Recent studies have shown a dose-dependent increase in dementia risk with one, two, and three co-morbid CMDs [5–8].

With no curative treatment for dementia available, it is crucial to identify factors that may offer protection against dementia risk. A large body of literature has related measures of mental and social stimulation – including high educational and occupational attainment, rich social network, engagement in leisure activities, minimal sedentary/screen-based time, and combinations thereof – to lower risk of dementia [9]. This may be explained by the concept of *cognitive reserve* (CR), the ability of lifelong engagement in cognitively stimulating activities to increase the adaptability of cognitive processes, thereby enhancing the brain’s capacity to withstand age- and pathology-related damage [10, 11].

The interplay between CR and risk factors for dementia, like CMDs, remains poorly understood. So far, previous studies from our group have linked lifestyles characterized by physical activity and social engagement to lower risk of dementia among people with T2D [12] and other CMDs [5].

Moreover, although CR is typically framed as the ability to cope with brain pathology (i.e. *resilience*), emerging evidence suggests that CR may additionally promote *resistance* – that is, reduced susceptibility to the accumulation of brain pathologies in the first place. In recent studies, CR-related factors have been related to lower white matter hyperintensity burden [13], reduced hippocampal atrophy [14], and a decrease in biomarkers of Alzheimer’s disease progression like cerebrospinal fluid Aβ42 and 18 F-FDG PET uptake [15]. To our knowledge, only one study has examined CR and resistance in the context of cardiometabolic health, finding that high education levels attenuated the association between hypertension and vascular brain damage [16].

The present study aims to comprehensively examine the interplay between CR and CMDs with respect to cognitive and brain aging. Using 15-year longitudinal data from > 200,000 older adults in the UK Biobank, including nearly 14,000 who underwent brain MRI, we examined the joint effect of CMDs and cognitive reserve on (1) dementia risk and (2) MRI markers of neurodegenerative and vascular brain damage.

## Methods

### Study design and population

The UK Biobank is an ongoing prospective longitudinal study of > 500,000 adults aged 40 to 70 recruited from across the United Kingdom [17]. The baseline examination was conducted between 2006 and 2010 and included sociodemographic, physical, and medical assessments. A subset of participants underwent a brain MRI scan between 2014 and 2020. Changes in health status were monitored for a maximum of 15 years (until January 2022) via linkage with medical records.

All participants provided informed consent at baseline and prior to the MRI scan. Data collection procedures have been approved by the UK National Research Ethics Service (Ref 11/NW/0382) in accordance with the Declaration of Helsinki.

Selection of the study population is illustrated in eFigure 1. Of 217,456 UK Biobank participants aged ≥ 60 at baseline, we excluded 166 with prevalent dementia and 926 with missing information on baseline CMDs. To avoid possible misclassification of the exposure, we additionally excluded 186 with type 1 diabetes, leaving a sample of 216,178. Of the 13,932 participants who underwent a brain MRI scan, we excluded 269 with chronic neurological diseases (eTable 1) to yield a neuroimaging subsample of 13,663.

### Assessment of cardiometabolic diseases

CMDs were defined as T2D, heart disease (including coronary heart disease, atrial fibrillation, and heart failure), and stroke [3] and were ascertained based on medical records, medication use, self-reported medical history, and biochemical measures (eTable 2). CMD status was dichotomized as the absence or presence of any CMD. CMD status was defined as participants’ total number of CMDs at baseline (0, 1, or ≥ 2) and dichotomized as CMD-free vs. CMDs.

### Cognitive reserve indicator

CR was defined based on information from the baseline examination about six reserve-related factors: higher education level, higher occupational attainment, more social contact, greater frequency of confiding in others, regular engagement in a greater number of leisure activities, and fewer hours spent watching television. These factors are frequently-used proxies of CR that encompass mental and social stimulation across early life (education [18, 19]), mid-life (occupation [20–22]), and late life (social and leisure activities [23–26]). Multiple items related to social and leisure activity were included in order to capture both the quantity (amount of social contact) and quality (frequency of confiding) of social relationships [27] as well as people’s level of engagement in both more-stimulating, active pursuits (leisure activities) as well as less-stimulating, passive ones (television watching) [28–30].

#### Education level

Participants self-reported their education level as one of the following: (1) no educational qualifications; (2) Certificate of Secondary Education or equivalent, O levels or equivalent; (3) National Vocational Qualification, Higher National Diploma, Higher National Certificate or equivalent; (4) A/AS levels or equivalent, other professional qualifications; or (5) college/university degree.

#### Occupational attainment

Participants provided details on their current (or, for retired individuals, longest-held) occupation, and this grouped into one of eight categories in the National Statistics Socio-economic Classification (NS-SEC), ranging from SEC-1 to SEC-8, where lower values reflect occupations with more required skills and training [31]. Occupational attainment was categorized as: (1) unemployed (SEC-8) or routine occupations (SEC-7); (2) semi-routine occupations (SEC-6), lower supervisory and technical occupations (SEC-5), or employers in small organizations (SEC-4); (3) intermediate occupations (SEC-3); (4) lower professional and higher technical occupations (SEC-2); or (5) higher professional occupations (SEC-1).

#### Social contact

Based on the question “How often do you visit friends or family or have them visit you?”, frequency of social contact was classified as: less than about once a month, about once per week, 2–4 times per week, or almost daily.

#### Confiding in others

Based on the question “How often are you able to confide in someone close to you?”, frequency of confiding in others was categorized as: never, around once per month, 1–4 times per week, or almost daily.

#### Leisure activities

Participants were presented with a list of social and leisure activities (sports club or gym, pub or social club, religious group, adult education class, or other group activity) and asked, “Which of the following do you attend once a week or more often?” Responses were classified as ≤ 1, 2, or 3–5 leisure activities per week.

#### Television watching time

Based on the question “In a typical day, how many hours do you spend watching television?”, television watching time was categorized as: ≥4, ≥3 to <4, ≥2 to <3, or <2 h/day.

Following previous studies from well-characterized cohorts like the Swedish National Study on Aging and Care, Kungsholmen (SNAC-K) [32, 33] and the Rush Memory and Aging Project (MAP) [34–36], we operationalized CR using a latent variable approach. Specifically, a CR indicator reflecting participants’ overall level of CR based on the six CR-related factors was generated using latent class analysis (LCA). LCA uses mixture modeling to identify hidden clusters by grouping multiple observed variables into a latent variable with mutually exclusive latent classes [37]. This approach therefore enabled us to capture a broad pattern of engagement in mentally and socially stimulating activities across the lifespan.

**Fig. 1:**
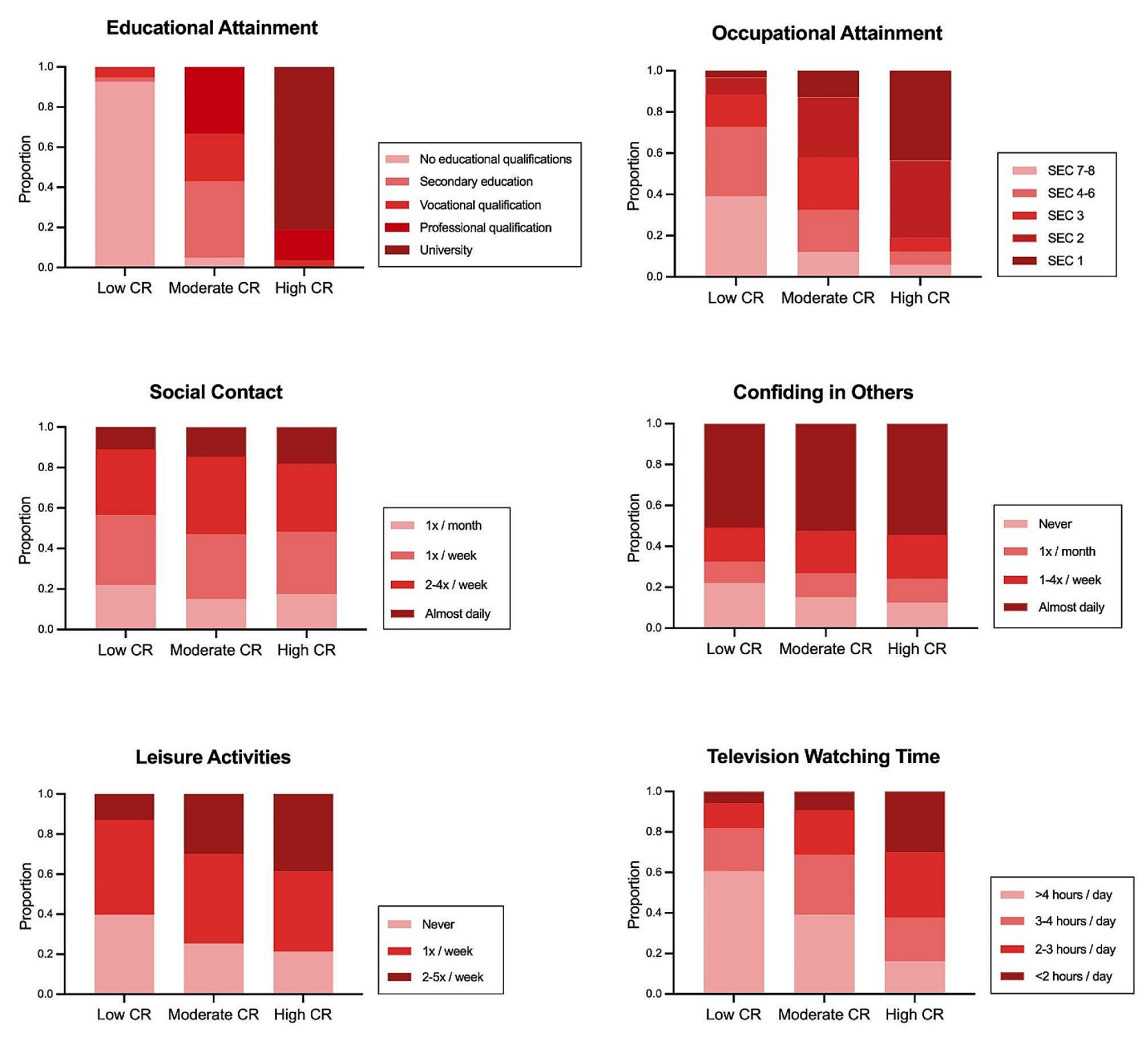
Distribution of cognitive reserve (CR)-related factors in the low, moderate, and high latent classes. Darker colors indicate more favorable and lighter colors indicate less favorable levels of each CR-related factor

Consistent with our previous work [38–41], a three-latent-class model was identified as having the best fit (eTable 3): *Latent class 1* (**high CR**) was majority college-educated and characterized by higher occupational attainment, more leisure activities, fewer daily hours of television, and moderate frequencies of confiding and social contact; *latent class 2* (**moderate CR**) was characterized by moderately favorable levels of all CR-related factors, and *latent class 3* (**low CR**) was comprised mostly of individuals who had completed secondary education or less and was characterized by lower occupational attainment, less engagement in leisure activities, more daily hours of television, and moderate frequencies of confiding and social contact (Fig. 1, eTable 4).

### Dementia diagnosis

Information from inpatient records, self-reported medical history, medication use, and death registers were algorithmically combined to identify dementia, including the Alzheimer’s disease (AD) and vascular dementia (VaD) subtypes [42]. The primary outcome of interest in this study was all-cause dementia; Alzheimer’s disease (AD) and vascular dementia (VaD) subtypes were considered as secondary outcomes.

### Brain MRI acquisition and pre-processing

A detailed description of the brain MRI image acquisition and processing protocols in the UK Biobank has been previously published [43–45]. Briefly, T1and T2 FLAIR imaging were performed to provide volumes of brain structures and white matter lesions, and diffusion tensor imaging (DTI) was used to estimate white matter microstructural integrity (eTable 5).

In this study, we examined volumes of gray matter (GMV) and hippocampus (HV) as markers of neurodegenerative brain damage. Vascular injury was assessed via white matter hyperintensity volume (WMHV) and as well as fractional anisotropy (FA) and mean diffusivity (MD) from DTI. GMV and HV were adjusted for intracranial volume. To enable comparison, GMV, HV, FA, and MD were converted to Z-scores. WMHV was log-transformed given its skewed distribution.

### Assessment of covariates

Socioeconomic status (SES) was assessed using Townsend Deprivation Indices (TDI), a measure of neighborhood-level socioeconomic deprivation [46]. Race and ethnicity were self-reported according to the 2001 UK census categories and dichotomized as white vs. non-white (including Asian, Black, multiracial, or other). Body mass index (BMI) was calculated based on baseline height and weight measurements and classified as underweight (< 20 kg/m^2^), normal (≥ 20 to < 25 kg/m^2^), overweight (≥ 25 to < 30 kg/m^2^), or obese (≥ 30 kg/m^2^). Hypertension was defined based on medical records, self-reported history of high blood pressure, antihypertensive medication use, or blood pressure measurement (systolic ≥ 140 mm Hg, diastolic ≥ 90 mm Hg). Smoking and drinking habits were self-reported as never, previous, or current. Physical activity was classified as inactive, moderate, or active based on the International Physical Activity Questionnaire [47]. Probable major depression was ascertained from items in the baseline questionnaire corresponding to the Structured Clinical Interview for *DSM-IV* Axis I Disorders [48]. *APOE* was genotyped from blood samples collected at baseline and dichotomized as carriers vs. non-carriers of the ε4 allele.

### Statistical analysis

Cox regression models were used to estimate the hazard ratios (HRs) and 95% confidence intervals (CIs) of dementia associated with CMDs and CR level. The timescale was defined as age at dementia diagnosis, death, or the last available follow-up (20 January 2022), whichever came first. The proportional hazard assumption was tested using Schoenfeld residuals. A violation of proportionality was observed for sex, so this was treated as a stratified factor in the model. We assessed the joint effect of CMDs and CR using a six-category indicator variable that combined CMD status (yes vs. no) and CR level (high, moderate, or low). Because similar results were found for the moderate and high CR groups, these were collapsed for ease of interpretation to yield a four-category indicator variable (reference: CMD-free/moderate-to-high CR). The difference in dementia risk between the CMD/moderate-to-high CR and CMD/low CR groups (i.e., HR_CMD & moderate−to−high CR_ / HR_CMD & low CR_) was statistically tested by repeating the models using the CMD/low CR group as the reference. Next, among participants with CMDs, we calculated the population attributable fraction (PAF) of CR for the risk of dementia (i.e., the proportion of dementia cases that could be avoided if all participants had high CR). Laplace regression was used to estimate the percentile differences (PDs) in time (in years) to dementia onset as a function of joint CMD and dietary inflammatory potential status. According to the cumulative incidence rate of dementia in this sample, the 5th PDs of dementia onset were estimated [49]. Finally, linear regression models were used to estimate β-coefficients and 95% CIs for the association of CMD status, CR level, and joint CMD/CR status with brain MRI measures (GMV, HV, WMHV, FA, and MD).

Multiplicative interactions were assessed by incorporating the CMD status × CR level cross-product term into the models. Additive interactions were assessed using relative excess risk due to interaction (RERI), attributable proportion (AP), and synergy index (S).

Models were first basic adjusted for socio-demographic factors (age, sex, SES, race/ethnicity) and next further adjusted for vascular risk factors (BMI, hypertension, smoking, drinking, physical activity), depression, and *APOE* ε4 carrier status. Neuroimaging analyses were additionally adjusted for assessment center and head and table position within the MRI scanner.

Missing values for CR-related factors (education [*n* = 4,228], occupation [*n* = 87,151], confiding [*n* = 8,383], social contact [*n* = 2,625], leisure activities [*n* = 713], and television watching [*n* = 2,128]) and covariates (SES [*n* = 184], race/ethnicity [*n* = 771], BMI [*n* = 1,129], hypertension [*n* = 583], smoking [*n* = 1,060], alcohol consumption [*n* = 226], physical activity [*n* = 46,698], and *APOE* ε4 [*n* = 38,937]) were imputed using fully conditional specification, with estimates pooled from 5 iterations.

Additional analyses were performed to identify possible sex differences (**Appendix A**) and to assess the contribution of each CR-related factor and each CMD separately (**Appendix B**). In sensitivity analyses, we (1) excluded dementia cases that occurred within the first 5 years of follow-up (*n* = 610) to minimize reverse causality; (2) used non-imputed data; and (3) accounted for the competing risk of death using Fine and Grey regression (**Appendix C**).

All analyses were performed using Stata SE 16.0 (StataCorp, College Station, TX). *P*-values < 0.05 were considered statistically significant.

## Results

### Baseline characteristics

Baseline characteristics of the 216,178 study participants (mean age 64.1 ± 2.9; 52.8% female) are described in Table 1. A total of 43,402 (20.1%) participants had at least one CMD at baseline. These individuals were more likely to be older, male, non-white, have lower SES, have a higher BMI, smoke, be physically inactive, have hypertension, and have low CR. They were less likely to drink alcohol or have depression.

**Table 1:** Baseline characteristics of the study population (*n* = 216,178)

Characteristics	Full Sample (*n* = 216,178)	By CMD status		
		CMD-free (*n* = 172,776)	CMDs (*n* = 43,402)	*P*-value
Age, years	64.1 ± 2.9	64.0 ± 2.8	64.7 ± 2.9	< 0.001
Sex				
Female	114,035 (52.8)	97,772 (56.6)	16,263 (37.5)	< 0.001
Male	102,143 (47.3)	75,004 (43.4)	27,139 (62.5)	
College/university-educated	56,128 (26.3)	47,194 (27.7)	8,934 (20.1)	< 0.001
White race/ethnicity	201,292 (93.5)	161,401 (93.7)	39,891 (92.3)	< 0.001
Townsend deprivation index	-1.6 ± 3.0	-1.7 ± 2.9	-1.0 ± 3.2	< 0.001
Body mass index (BMI), kg/m^2^	27.6 ± 4.6	27.1 ± 4.3	29.6 ± 5.1	< 0.001
Underweight (< 20)	3,961 (1.8)	3,590 (2.1)	371 (0.9)	< 0.001
Normal (20–25)	60,165 (28.0)	53,337 (31.0)	6,828 (15.9)	
Overweight (25–30)	97,123 (45.2)	78,888 (45.9)	18,235 (42.4)	
Obese (≥ 30)	53,800 (25.0)	36,219 (21.0)	17,581 (40.1)	
Smoking				
Never	107,472 (50.0)	89,665 (52.1)	17,807 (41.3)	< 0.001
Previous	89,820 (41.8)	68,803 (40.0)	21,017 (48.8)	
Current	17,826 (8.3)	13,573 (7.9)	4,253 (9.9)	
Alcohol				
Never	10,359 (4.8)	7,524 (4.4)	2,835 (6.5)	< 0.001
Previous	8,246 (3.8)	5,579 (3.2)	2,667 (6.15)	
Current	197,347 (91.4)	159,514 (92.4)	37,833 (87.3)	
Physical activity				
Low	29,210 (17.2)	21,632 (15.9)	7,578 (22.6)	< 0.001
Moderate	70,951 (41.9)	57,124 (42.0)	13,827 (41.3)	
High	69,319 (40.1)	57,236 (42.1)	12,083 (36.1)	
Hypertension	82,950 (38.5)	55,881 (32.3)	27,069 (63.2)	< 0.001
Depression	35,825 (16.6)	29,961 (17.3)	5,864 (13.5)	< 0.001
*APOE* ε4 carrier	50,199 (28.3)	40,252 (28.3)	9,947 (28.6)	0.230
Cognitive reserve (CR) indicator				
Low	58,978 (27.3)	43,545 (25.2)	15,433 (35.6)	< 0.001
Moderate	87,271 (40.4)	70,493 (40.8)	16,778 (38.7)	
High	69,929 (32.4)	58,738 (34.0)	11,191 (25.8)	

Data are presented as means ± standard deviations or number (proportion, %)

Missing data: 2,907 for education level; 771 for race/ethnicity; 184 for Townsend deprivation index; 1,129 for BMI; 1,060 for smoking status; 226 for alcohol drinking; 46,698 for physical activity level; 583 for hypertension; 38,937 for APOE ε4 status

The neuroimaging subsample (*n* = 13,663) was comparatively younger with a higher SES and more favorable vascular risk factor profile (eTable 6–7).

### CMDs, CR, and dementia

Over the follow-up (median 11.7 years), a total of 6,600 (3.1%) participants developed dementia, including 2,866 (1.4%) with AD and 1,547 with VaD (0.7%). HRs for the association of CMD status and CR level with dementia are presented in Table 2. Having CMDs was associated with significantly increased risk of all-cause dementia (HR 1.74 [95% CI 1.65, 1.84]), AD (1.53 [1.40, 1.67]), and VaD (2.41 [2.17, 2.69]), and the risk of dementia and its subtypes increased dose-dependently with the presence of a greater number of CMDs. On the other hand, compared to low CR, moderate and high CR were related to a 15% (HR 0.85 [0.80, 0.90]) and 25% (HR: 0.75 [0.70, 0.80]) lower risk of dementia, respectively. Similar results were observed for AD and VaD.

**Table 2:** Associations of cardiometabolic diseases (CMDs) and cognitive reserve (CR) level with incident dementia

CMD/CR status	No. of subjects	All-cause dementia (*n* = 6,600 cases)			Alzheimer’s disease (*n* = 2,866 cases)			Vascular dementia (*n* = 1,547 cases)	
		Basic Adjusted HR (95% CI)	Multi-Adjusted HR (95% CI)		Basic Adjusted HR (95% CI)	Multi-Adjusted HR (95% CI)		Basic Adjusted HR (95% CI)	Multi-Adjusted HR (95% CI)
**CMD status**									
CMD-free	172,776	Reference	Reference		Reference	Reference		Reference	Reference
CMDs	43,402	**1.81 (1.72–1.91)**	**1.74 (1.65–1.84)**		**1.50 (1.39–1.63)**	**1.53 (1.40–1.67)**		**2.78 (2.51–3.09)**	**2.41 (2.17–2.69)**
1 CMD	36,441	**1.62 (1.53–1.72)**	**1.57 (1.48–1.67)**		**1.40 (1.28–1.53)**	**1.42 (1.30–1.56)**		**2.26 (2.01–2.53)**	**2.02 (1.79–2.28)**
≥2 CMDs	6,961	**2.88 (2.63–3.15)**	**2.79 (2.54–3.07)**		**2.11 (1.81–2.45)**	**2.24 (1.91–2.62)**		**5.67 (4.88–6.59)**	**4.79 (4.08–5.62)**
*Greater number of CMDs*		**1.65 (1.59–1.71)**	**1.63 (1.57–1.70)**		**1.42 (1.33–1.51)**	**1.47 (1.37–1.57)**		**2.29 (2.15–2.45)**	**2.15 (1.99–2.32)**
**CR level**									
Low CR	58,978	Reference	Reference		Reference	Reference		Reference	Reference
Moderate CR	87,271	**0.79 (0.74–0.83)**	**0.85 (0.80–0.90)**		**0.80 (0.73–0.87)**	**0.85 (0.78–0.93)**		**0.70 (0.62–0.78)**	**0.77 (0.69–0.87)**
High CR	69,929	**0.65 (0.61–0.69)**	**0.75 (0.70–0.80)**		**0.65 (0.59–0.72)**	**0.73 (0.66–0.80)**		**0.48 (0.42–0.55)**	**0.59 (0.52–0.68)**
*Higher CR level*		**0.80 (0.78–0.83)**	**0.86 (0.84–0.89)**		**0.81 (0.77–0.85)**	**0.85 (0.81–0.90)**		**0.69 (0.65–0.74)**	**0.77 (0.72–0.83)**

Basic-adjusted models included age at baseline, sex, race/ethnicity, and socioeconomic status. Multi-adjusted models additionally included body mass index, smoking status, alcohol drinking, physical activity, hypertension, depression, and *APOE* ε4 carrier status

**Fig. 2:**
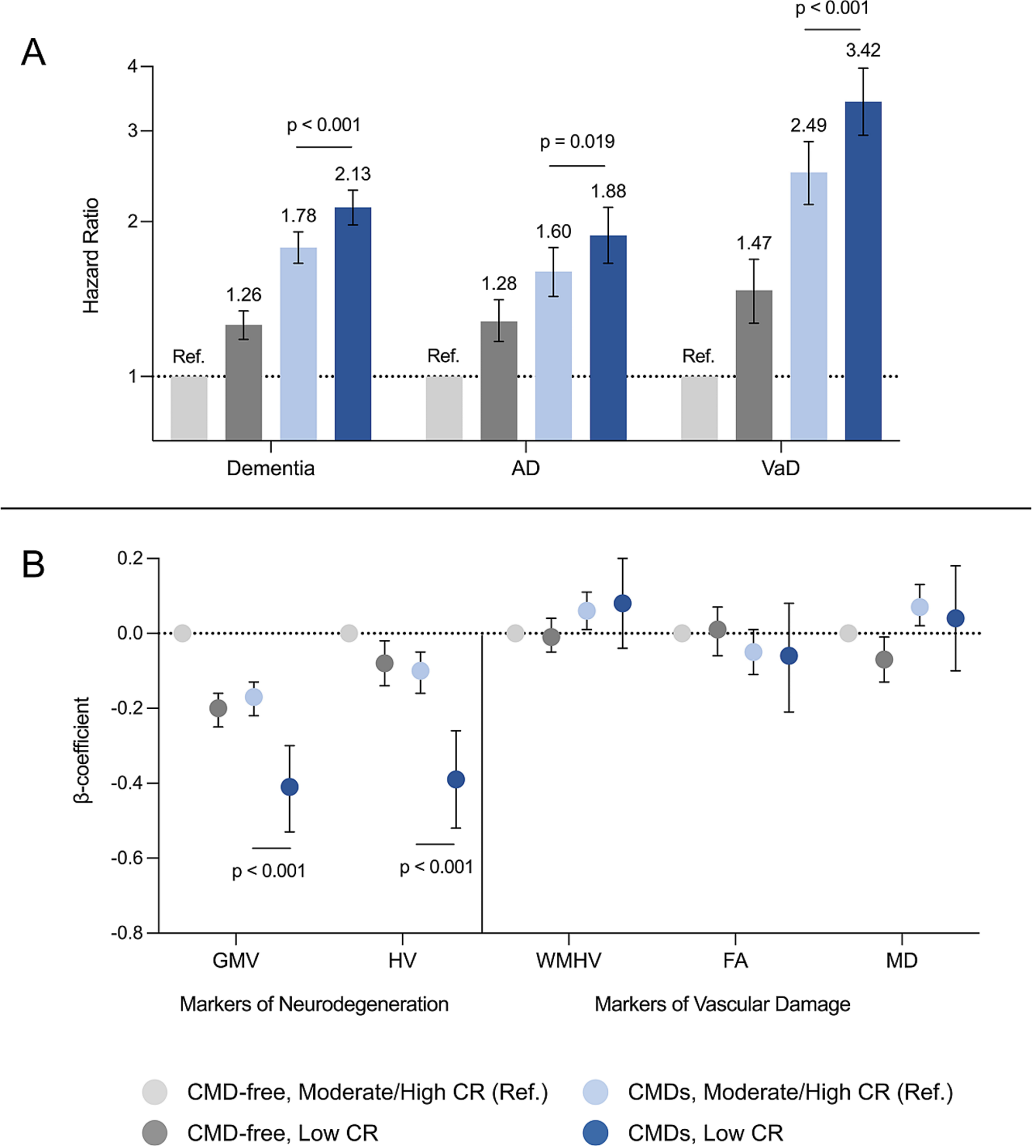
Joint effect of cardiometabolic disease (CMD) status and cognitive reserve (CR) level on dementia risk and neuroimaging measures. Panel A: Results from Cox regression models adjusted for age at baseline, sex, race/ethnicity, socioeconomic status, body mass index, smoking status, alcohol drinking, physical activity, hypertension, depression, and *APOE* ε4 carrier status. Panel B: Results from linear regression models additionally adjusted for MRI-reflated factors (head position, scanner table position, and assessment center). In all models, significant differences between the CMD/moderate-to-high CR and CMD/low CR groups were assessed by repeating the models using the CMD/low CR group as the reference. Abbreviations: AD = Alzheimer’s disease; VaD = vascular dementia; TBV = total brain volume; GMV = gray matter volume; WMV = white matter volume; HV = hippocampal volume; WMHV = white matter hyperintensity volume

In joint effect analysis, the HR of dementia was 2.32 (2.12, 2.54) for those with CMDs and low CR, 1.99 (1.81, 2.19) for those with CMDs and moderate CR, and 1.84 (1.65, 2.05) for those with CMDs and high CR (reference: CMD-free, high CR) (Table 3). Among people with CMDs, having moderate-to-high compared to low CR was associated with 17% lower risk of dementia (HR 0.83 [0.77, 0.91], *p* < 0.001; Fig. 2A). 15% lower risk of AD (HR 0.84 [0.74, 0.97], *p* = 0.019) and 27% lower risk of VaD (HR 0.73 [0.63, 0.85], *p* < 0.001) were also observed (Fig. 2A). Among people with CMDs, the proportion of dementia cases attributable to low CR was 0.11 (0.04, 0.18). In Laplace regression, participants with CMDs and high CR developed dementia 0.88 years later than those with CMDs and low CR (-1.70 vs. -2.58 years) (Table 3). However, no significant additive (RERI: 0.09 [-0.09, 0.26], AP: 0.04 [-0.04, 0.13], S: 1.09 [0.92, 1.29]) or multiplicative (*p* = 0.340) interactions between CMD status and CR level were observed.

**Table 3:** Joint effect of cardiometabolic diseases (CMDs) and cognitive reserve (CR) level: results from Cox and Laplace regression models

Joint Exposure		No. of subjects	Hazard ratio of dementia (95% CI)			Difference in time (years) to dementia diagnosis (95% CI)			Population attributable fraction (95% CI)
CMDs	CR level		Basic Adjusted	Multi-Adjusted		Basic Adjusted	Multi-Adjusted		
CMD-free	High	58,738	Reference	Reference		Reference	Reference		
CMD-free	Moderate	70,493	**1.20 (1.11–1.29)**	**1.15 (1.07–1.24)**		**-0.46 (-0.66**,** -0.26)**	**-0.37 (-0.59**,** -0.16)**		
CMD-free	Low	43,545	**1.49 (1.38–1.61)**	**1.37 (1.27–1.48)**		**-1.05 (-1.28**,** -0.82)**	**-0.86 (-1.10**,** -0.62)**		
CMDs	High	11,191	**1.90 (1.70–2.11)**	**1.83 (1.64–2.04)**		**-1.64 (-1.94**,** -1.35)**	**-1.70 (-2.06 -1.34)**		**0.11 (0.04–0.18)**
CMDs	Moderate	16,778	**2.13 (1.94–2.33)**	**1.97 (1.80–2.17)**		**-2.16 (-2.49**,** -1.84)**	**-2.03 (-2.38**,** -1.67)**		
CMDs	Low	15,433	**2.58 (2.37–2.82)**	**2.30 (2.10– 2.52)**		**-2.85 (-3.16**,** -2.54)**	**-2.58 (-2.89**,** -2.26)**		

Basic-adjusted models included age at baseline, sex, race/ethnicity, and socioeconomic status. Multi-adjusted models additionally included body mass index, smoking status, alcohol drinking, physical activity, hypertension, depression, and *APOE* ε4 carrier status

### CMDs, CR, and brain MRI measures

β-coefficients for the association of CMD status and CR level on brain MRI measures are presented in Table 4. CMDs were associated with markers of both neurodegenerative and vascular damage, including significantly smaller GMV (β -0.18 [-0.22, -0.13]) and HV (β -0.13 [-0.18, -0.08]) as well as significantly larger WMHV (β 0.06 [0.02, 0.11]) and MD (β 0.08 [0.02, 0.13]). On the other hand, compared to low CR, moderate and high CR were associated with significantly larger GMV and HV, but no difference in markers of vascular damage.

**Table 4:** Association of cardiometabolic diseases (CMD) status and cognitive reserve (CR) level with neuroimaging measures

CMD and CR status		No. of subjects	Markers of Neurodegeneration			Markers of Vascular Damage		
			Gray Matter Volume	Hippocampal Volume		White Matter Hyperintensity Volume	Fractional Anisotropy	Mean Diffusivity
			β (95% CI)	β (95% CI)		β (95% CI)	β (95% CI)	β (95% CI)
**CMD status**								
CMD-free		11,880	Reference	Reference		Reference	Reference	Reference
CMDs		1,783	**-0.18 (-0.22**,** -0.13)**	**-0.13 (-0.18**,** -0.08)**		**0.06 (0.02**,** 0.11)**	-0.05 (-0.11, 0.01)	**0.08 (0.02**,** 0.13)**
**CR level**								
Low CR		1,499	Reference	Reference		Reference	Reference	Reference
Moderate CR		5,147	**0.13 (0.08**,** 0.18)**	**0.06 (0.01**,** 0.12)**		0.00 (-0.05, 0.05)	-0.03 (-0.09, 0.03)	0.05 (-0.02, 0.11)
High CR		7,017	**0.27 (0.22**,** 0.32)**	**0.14 (0.09**,** 0.20)**		0.00 (-0.05, 0.05)	0.02 (-0.04, 0.08)	0.05 (-0.01, 0.11)
**Joint Exposure**								
*CMDs*	*CR level*							
CMD-free	High	6,171	Reference	Reference		Reference	Reference	Reference
CMD-free	Moderate	4,430	**-0.13 (-0.16**,** -0.09)**	**-0.07 (-0.11**,** -0.03)**		0.00 (-0.03, 0.04)	**-0.05 (-0.09**,** -0.01)**	0.01 (-0.03, 0.05)
CMD-free	Low	1,279	**-0.26 (-0.31**,** -0.21)**	**-0.11 (-0.17**,** -0.05)**		0.00 (-0.06, 0.05)	-0.02 (-0.08, 0.04)	**-0.06 (-0.13**,** -0.01)**
CMDs	High	846	**-0.14 (-0.20**,** -0.08)**	**-0.07 (0.14**,** -0.01)**		**0.07 (0.00**,** 0.13)**	-0.06 (-0.14, 0.02)	**0.10 (0.02**,** 0.17)**
CMDs	Moderate	717	**-0.33 (-0.39**,** -0.26)**	**-0.21 (-0.29**,** -0.14)**		0.05 (-0.01, 0.12)	**-0.09 (-0.18**,** -0.01)**	0.06 (-0.03, 0.14)
CMDs	Low	220	**-0.47 (-0.58**,** -0.36)**	**-0.42 (-0.55**,** -0.29)**		0.08 (-0.04, 0.20)	-0.09 (-0.23, 0.06)	0.05 (-0.19, 0.20)

All models were adjusted for age at baseline, sex, race/ethnicity, socioeconomic status, body mass index, smoking status, alcohol drinking, physical activity, hypertension, depression, APOE ε4 carrier status, and MRI-reflated factors (head position, scanner table position, and assessment center)

In joint effect analysis, higher CR levels appeared to attenuate the association between CMDs and MRI markers of neurodegenerative, but not vascular, damage. Participants with CMDs and moderate-to-high compared to low CR had significantly larger GMV (β -0.17 [-0.22, -0.13] vs. β -0.41 [-0.53, -0.30]) and HV (β -0.10 [-0.16, -0.05] vs. β -0.39 [0.52, -0.26]), but similar WMHV, FA, and MD (Fig. 2B). We detected a significant multiplicative interaction between CMD status and CR level for GMV (*p* < 0.001) and HV (*p* < 0.001).

### Additional analyses

**Fig. 3:**
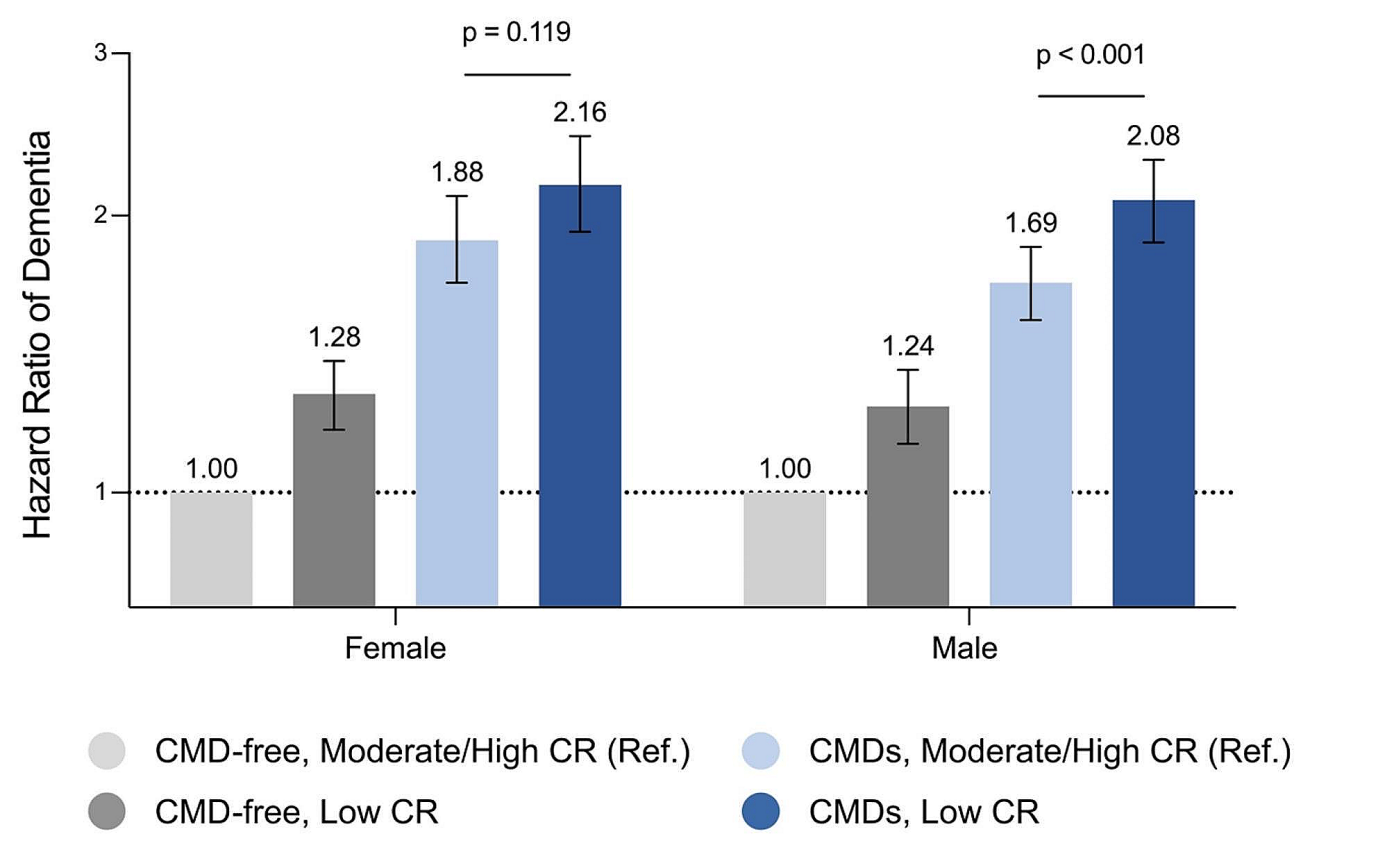
Sex differences in the relationship between cardiometabolic diseases (CMD) and cognitive reserve (CR) level on dementia risk. Results from Cox regression models adjusted for age at baseline, race/ethnicity, socioeconomic status, body mass index, smoking status, alcohol drinking, physical activity, hypertension, depression, and *APOE* ε4 carrier status. For sex-stratified analyses, CR was calculated separately in males and females (eFigure 2). See eTable 8–9 for additional results. Significant differences between the CMD/moderate-to-high CR and CMD/low CR groups were assessed by repeating the models using the CMD/low CR group as the reference.

In sex-stratified analyses (Fig. 3), dementia risk was significantly lower in males with CMDs and moderate-to-high vs. low CR (HR 0.81 [0.73, 0.90], *p* < 0.001); no significant difference in dementia risk was detected between females with CMDs and moderate-to-high vs. low CR (HR 0.90 [0.78, 1.03], *p* = 0.119) (eFigure 2; eTable 8–9). Additional analyses considering each individual CR-related factor and each CMD separately are described in eTable 10–12. In sensitivity analysis, similar results were obtained when we repeated the analyses using non-imputed data (eTable 13), after excluding dementia cases that occurred within the first 5 years of follow-up (eTable 14), and after accounting for the competing risk of death (eTable 15).

## Discussion

In this large-scale study community-based study of over 200,000 participants, we found that people with CMDs and moderate-to-high compared to low levels of CR had (1) 17% lower risk of dementia and (2) significantly larger gray matter and hippocampal volumes.

Our study adds to a growing literature highlighting the relationship between CMDs and increased risk of dementia [6–8]. A relevant issue is the identification of modifiable factors that could help older adults with CMDs maintain cognitive health. Recent studies from our group have linked active life (characterized by high physical activity and social integration) to lower risk of dementia in people with T2D [12] and other CMDs [5], but the specific role of mental and social activity has so far been unaddressed.

In this study, we investigated whether a comprehensive indicator of CR – integrating educational and occupational attainment, social network, and leisure activities – can attenuate the risk of dementia associated with CMDs. Over 15 years of follow-up, dementia risk was 17% lower among participants with CMDs and moderate-to-high compared to low levels of CR. We further estimated that people with CMDs and high CR developed dementia nearly 1 year later than those with low CR and that 11% of dementia cases among people with CMDs could be avoided with the adoption of a lifestyle characterized by high CR. In addition, the risk of AD and VaD were 15% and 27% lower among people with CMDs and moderate-to-high vs. low CR, suggesting that CR may compensate against a variety of underlying etiologies.

CR is hypothesized to protect against cognitive decline and dementia by increasing the brain’s *resilience* to damage through neural reserve (i.e. higher intrinsic brain network connectivity that is better able to tolerate brain pathology without experiencing cognitive impairment) [50] and/or neural compensation (i.e. fostering the development of alternative neural pathways that take over the tasks performed by damaged ones) [51]. In this study, we examined whether CR may be additionally related to *resistance* to the development of brain pathology in the first place. We found that people with CMDs and moderate-to-high compared to low CR had significantly larger GMV and HV, but no difference in WMHV, FA, or MD. This could indicate that CR may offer some protection against gray matter and hippocampal atrophy in people with CMDs, but not the accumulation of vascular pathologies. In support of this, a recent systematic review and meta-analysis of longitudinal brain MRI studies concluded that higher cognitive/social engagement is associated with reduced hippocampal atrophy in older age, but no clear trend in the accumulation of WMHs or changes in DTI measures [52]. On the other hand, a recent investigation reported that higher levels of education attenuated the association between cardiovascular risk score and vascular pathologies including WMHs and lacunes [16].

Several potential mechanisms could explain the observed association of CR with lower dementia risk and larger GMV and HV. On one hand, engagement in mentally and socially stimulating activities might reduce systemic inflammation, oxidative stress, and other biological pathways that drive brain and cognitive aging [53]. Additionally, people with high levels of CR may have greater socioeconomic advantages, richer social resources, and higher health literacy, making it easier to adhere to a healthy lifestyle and manage risk factors for dementia. Alternatively, it could be that having lower levels of brain pathology facilitates engagement in a mentally- and socially- active life in the first place. Longitudinal brain MRI studies are needed to better understand the interaction between CR and brain pathology, especially in the context of other risk factors like CMDs.

Adding to this complexity, sex may play a role in the relationship between CMDs, CR, and dementia. In sex-stratified analyses, dementia risk was 19% lower in males with CMDs and moderate-to-high vs. low CR, but no significant difference was observed in females. Future studies are warranted to explore possible biological (ex. sex differences in the prevalence and severity of CMDs) [54] as well as sociological (ex. gender-based disparities in important CR-related factors like educational/occupational attainment) [55] explanations for these findings.

Another remaining question is which sources of CR are driving the attenuation of dementia risk among people with CMDs. Our operationalization of CR using LCA was intended to capture not individual CR-related items but rather the underlying correlation and complex interplay of these (often overlapping) factors. However, in joint effect analyses comparing dementia risk between people with CMDs and the most compared to the least favorable levels of each individual CR-related factor (eTable 11), the largest attenuation in dementia risk was observed for higher occupational attainment (26%, *p* < 0.001) followed by greater engagement in leisure activities (25%, *p* < 0.001), fewer daily hours of television (24%, *p* = 0.001), more frequent confiding (21%, *p* < 0.001), higher education (16%, *p* = 0.006), and more social contact (11%, *p* = 0.091). The lower dementia risk associated with late-life behaviors like engagement in leisure activities, minimal television watching, and confiding in others is encouraging and highlights that it is never too late for older individuals to implement lifestyle changes that may increase CR.

### Strengths and limitations

Strengths of this study lie in the use of a large-scale population-based study with a comprehensive data collection procedure, including brain MRI scans for > 13,000 participants. However, our findings should be considered in the context of several limitations. First, as both dementia and CMDs were ascertained primarily via medical records, it is likely that some cases went undetected. Differential outcome misclassification is possible insofar as people with diagnosed CMDs may interact more with the healthcare system and therefore be more likely receive a dementia diagnosis. However, the magnitude of the CMD-dementia association reported here was similar to what has been observed in previous studies where both CMDs and dementia were diagnosed through regular physician examinations [5, 6]. Another limitation is that brain MRI scans were conducted at only one time point, so the relationship between CR, CMDs, and changes in brain MRI phenotypes could not be examined. This may be possible in future studies, as collection of repeat brain MRI scans is currently ongoing among a subset of approximately 10,000 UK Biobank participants [56]. Finally, the UK Biobank suffers from well-documented healthy volunteer bias [57, 58], which could limit the generalizability of our findings and may have contributed to an underestimation of the observed associations. Selection bias may be stronger in the neuroimaging subsample given the 9-year time interval between baseline and the MRI scan (i.e., survival bias).

## Conclusions

Among people with CMDs, having a higher level of CR was associated with lower dementia risk and larger gray matter and hippocampal volumes. These results highlight a mentally and socially active life as a modifiable factor that may support cognitive and brain health among people with CMDs.

### Electronic supplementary material

Below is the link to the electronic supplementary material.

Supplementary Material 1

## Data Availability

UK Biobank data is not publicly available, but researchers can apply for access here: https://www.ukbiobank.ac.uk/enable-your-research/apply-for-access.

## Abbreviations

AD: Alzheimer’s disease
AP: Attributable proportion
BMI: Body mass index
CMDs: Cardiometabolic diseases
CR: Cognitive reserve
DTI: Diffusion tensor imaging
FA: Fractional anisotropy
GMV: Gray matter volume
HV: Hippocampal volume
LCA: Latent class analysis
MD: Mean diffusivity
MRI: Magnetic resonance imaging
NS-SEC: National Statistics Socio-economic Classification
PAF: Population attributable fraction
PPV: Positive predictive value
RERI: Relative excess risk due to interaction
S: Synergy index
SES: Socioeconomic status
T2D: Type 2 diabetes
VaD: Vascular dementia
WMHV: White matter hyperintensity volume
