# Supplementary material for: High cognitive reserve attenuates the risk of dementia associated with cardiometabolic diseases
Source: Alzheimers Res Ther. 2024 Jul 19;16:161. doi: 10.1186/s13195-024-01528-2 (PMC11264799; doi:10.1186/s13195-024-01528-2)
Supplement: Supplementary file 1 — Supplementary Material 1 [file 13195_2024_1528_MOESM1_ESM.docx]

**SUPPLEMENTARY MATERIALS**

**eFigure 1.** Flow chart of the study population.

**eTable 1.** Neurological disorders used as exclusion criteria for the neuroimaging subsample.

**eTable 2.** UK Biobank Field Codes used to ascertain baseline CMD status.

**eTable 3.** *G*^2^ statistics, Akaike Information Criterion, Bayesian information criterion, and mean posterior of different solutions from latent class analysis.

**eTable 4.** Comparison of baseline characteristics in the low, moderate, and high CR latent classes.

**eTable 5.** UK Biobank brain MRI image acquisition protocols.

**eTable 6.** Baseline characteristics of the neuroimaging study population.

**eTable 7.** Comparison of baseline characteristics among participants included vs. not included the neuroimaging subsample.

**APPENDIX A: Sex Differences**

**eFigure 2.** Distribution of cognitive reserve (CR)-related factors in the sex-specific low, moderate, and high latent classes.

**eTable 8.** Summary of main results for females.

**eTable 9.** Summary of main results for males.

**APPENDIX B: Analyses considering CR-related factors and CMDs separately**

**eTable 10.** Hazard ratios (HR) and from Cox regression models for the association between CR-related factors and incident dementia.

**eTable 11.** Hazard ratios (HR) and from Cox regression models for the joint effect of CMD status and CR components on risk of incident dementia.

**eTable 12.** Hazard ratios (HR) from Cox regression models for the joint effect of individual CMDs and CR on risk of incident dementia.

**APPENDIX C: Sensitivity Analyses**

**eTable 13.** Summary of main results using non-imputed data for covariates.

**eTable 14.** Hazard ratios (HR) from Cox regression models for the associations of CMD status and CR level with dementia risk, after excluding 610 study participants who developed dementia within the first 5 years of follow-up (n=215,568).

**eTable 15.** Sub-distribution hazard ratios (SHR) from Fine & Gray regression models for the associations of CMD status and CR level with dementia risk, accounting for the competing risk of death.

**eFigure 1. Flow chart of the study population.**

**217,456 aged ≥60 at baseline**

**Exclude:**

- **166 with prevalent dementia**
- **186 with type 1 diabetes**
- **926 with missing information on baseline CMDs (T2D, HD, or stroke)**

**Exclude:**

- **202,246 who did not undergo brain MRI scan**
- **269 with chronic neurological diseases**

**502,365 participants in the UK Biobank**

**216,178 included in the analysis of incident dementia**

**13,663 included in the neuroimaging subsample**

**(brain MRI scans conducted 9 years after baseline)**

**eTable 1. Neurological disorders used as exclusion criteria for the neuroimaging subsample.**

| **Neurological disorder (self-reported)** | **Code (Field ID 20002 and 20003)** |
| --- | --- |
| Parkinson’s disease | 1262 |
| Dementia or Alzheimer’s disease | 1263 |
| Chronic degenerative neurological | 1258 |
| Guillain-Barré syndrome | 1256 |
| Multiple Sclerosis | 1261 |
| Other demyelinating disease | 1397 |
| Brain cancer | 1032 |
| Brain/intracranial abscess | 1245 |
| Cerebral aneurysm | 1425 |
| Cerebral palsy | 1433 |
| Encephalitis | 1246 |
| Epilepsy | 1264 |
| Head injury | 1266 |
| Infections of the nervous system | 1244 |
| Meningeal cancer | 1031 |
| Meningioma (benign) | 1659 |
| Meningitis | 1247 |
| Motor Neuron Disease | 1259 |
| Neurological injury/trauma | 1240 |
| Spina bifida | 1524 |
| Subdural hematoma | 1083 |

**eTable 2. UK Biobank Field Codes used to ascertain baseline CMD status.**

|  | **Medical Records (ICD-10 codes)** | **Self-Reported Medical History** | **Self-Reported Medications** | **Biochemical Measures** |
| --- | --- | --- | --- | --- |
| **Type 2 diabetes** | 130709 (E11)  130711 (E12)  130713 (E13)  130715 (E14) | 2443  2976 | 6153  6177 | 30750 (HbA1c ≥6.5%)  30740 (FPG ≥126 mg/dl) |
| **Heart disease** | 131297 (I20)  131299 (I21)  131301 (I22)  131303 (I23)  131305 (I24)  131307 (I25)  131351 (I48)  131353 (I49)  131355 (I50) | 3627  3894  6150 |  |  |
| **Stroke** | 131361 (I60)  131363 (I61)  131365 (I62)  131367 (I63)  131375 (I67)  131367 (I68) | 4056 |  |  |

**eTable 3. *G*^2^ statistics, Akaike Information Criterion, Bayesian information criterion, and mean posterior of different solutions from latent class analysis.**

| Models | *G*^2^ | Akaike Information Criterion | Bayesian information criterion | Mean posterior probabilities | | | | | |
| --- | --- | --- | --- | --- | --- | --- | --- | --- | --- |
|  |  |  |  | Latent class 1 | Latent class 2 | Latent class 3 | Latent class 4 | Latent class 5 | Latent class 6 |
| One-latent-class model | 106306.17 | 106344.17 | 106539.56 | 1.00 | - | - | - | - | - |
| Two-latent-class model | 26709.30 | 26787.30 | 27188.37 | 0.90 | 0.90 | - | - | - | - |
| Three-latent-class model | 17203.26 | 17321.26 | 17928.00 | 0.83 | 0.87 | 0.76 | - | - | - |
| Four-latent-class model | 12805.25 | 12963.25 | 13775.68 | 0.80 | 0.60 | 0.72 | 0.81 | - | - |
| Five-latent-class model | 10367.31 | 10565.31 | 11583.42 | 0.77 | 0.67 | 0.65 | 0.63 | 0.61 | - |
| Six-latent-class model | 9186.60 | 9424.60 | 10648.39 | 0.72 | 0.65 | 0.66 | 0.63 | 0.59 | 0.64 |

*G*^2^ statistics and Bayesian information criterion decreased as the number of latent classes grew from one to six. However, some of the mean posterior probabilities were <0.70 when the number of latent classes exceeded three. As a result, the three-latent-class model was identified as having the best balance between model selection and the uncertainty of posterior classification.

**eTable 4. Comparison of baseline characteristics in the low, moderate, and high CR latent classes.**

| **Characteristics** | **Low CR**  (n=58,978) | **Moderate CR**  (n=87,271) | **High CR**  (n=69,929) | **P-value** |
| --- | --- | --- | --- | --- |
| Age, years | 64.6 ± 2.9 | 64.1 ± 2.8 | 63.8 ± 2.8 | <0.001 |
| Sex |  |  |  |  |
| Female | 31,961 (54.2) | 49,142 (56.3) | 32,932 (47.1) | <0.001 |
| Male | 27,017 (45.8) | 38,129 (43.7) | 36,997 (52.9) |  |
| White | 55,923 (95.0) | 82,131 (94.4) | 63,238 (90.1) | <0.001 |
| Townsend deprivation index | -0.7 ± 3.3 | -1.9 ± 2.8 | -1.9 ± 2.8 | <0.001 |
| Body mass index (BMI), kg/m^2^ | 28.5 ± 4.8 | 27.7 ± 4.5 | 26.8 ± 4.2 | <0.001 |
| Underweight (<20) | 827 (1.4) | 1,479 (1.7) | 1,655 (2.4) | <0.001 |
| Normal (20 – 25) | 12,904 (22.0) | 23,595 (27.2) | 23,666 (34.0) |  |
| Overweight (25 – 30) | 26,207 (44.8) | 39,607 (45.6) | 31,309 (45.0) |  |
| Obese (≥30) | 18,626 (31.8) | 22,206 (25.6) | 12,968 (18.6) |  |
| Smoking |  |  |  |  |
| Never | 25,731 (44.0) | 43,329 (49.8) | 38,412 (55.1) | <0.001 |
| Previous | 25,632 (43.9) | 36,877 (42.4) | 27,311 (39.2) |  |
| Current | 7,075 (12.1) | 6,732 (7.7) | 4,019 (5.8) |  |
| Alcohol |  |  |  |  |
| Never | 3,925 (6.7) | 3,993 (4.6) | 2,441 (3.5) | <0.001 |
| Previous | 3,450 (5.9) | 2,816 (3.2) | 1,980 (2.8) |  |
| Current | 51,499 (87.5) | 80,395 (92.2) | 65,453 (93.7) |  |
| Physical activity |  |  |  |  |
| Low | 7,850 (19.0) | 11,605 (16.8) | 9,755 (16.5) | <0.001 |
| Moderate | 16,066 (38.9) | 28,636 (41.5) | 26,249 (44.3) |  |
| High | 17,360 (42.1) | 28,725 (4.7) | 23,234 (39.2) |  |
| Hypertension | 25,275 (43.1) | 33,246 (38.2) | 24,429 (35.0) | <0.001 |
| Depression | 5,195 (8.8) | 14,184 (16.3) | 16,446 (23.5) | <0.001 |
| *APOE* ε4 carrier | 13,522 (28.5) | 20,300 (28.3) | 16,377 (28.2) | 0.498 |
| CMDs | 15,433 (26.2) | 16,778 (19.2) | 11,191 (16.0) | <0.001 |

Data are presented as means ± standard deviations or number (proportion, %).

Missing data: 771 for race/ethnicity; 184 for Townsend deprivation index; 1,129 for BMI; 1,060 for smoking status; 226 for alcohol drinking; 46,698 for physical activity level; 583 for hypertension; 38,937 for APOE ε4 status.

**eTable 5. UK Biobank brain MRI image acquisition protocols.**

| **Modality** | **Duration** | **Voxel, Matrix** | **Key Parameters** |
| --- | --- | --- | --- |
| T1 | 5 minutes | 1×1×1 mm  208×256×256 | 3D MPRAGE, sagittal, R=2, TI/TR=880/2000 ms |
| T2 FLAIR | 6 minutes | 1.05×1.0×1.0 mm  192×256×256 | FLAIR, 3D SPACE, sagittal, R=2, PF 7/8, fat sat, TI/TR=1800/5000 ms, elliptical |
| DTI | 7 minutes | 2.0×2.0×2.0 mm  104×104×72 | MB=3, R=1, TE/TR=92/3600 ms, PF 6/8, fat sat, b=0 s/mm^2^ (5x + 3×phase-encoding reversed), b=1 000 s/mm^2^ (50×), b=2000 s/mm^2^ (50×) |

Abbreviations: R, in-plane acceleration factor; MB, multiband factor; PF, partial Fourier.

All non-EPI scans are pre-scan normalized (on-scanner bias-field corrected). Gradient distortion correction is deselected on the scanner and applied in post-processing.

**eTable 6. Baseline characteristics of the neuroimaging study population.**

| **Characteristics** | **Overall**  (n=13,663) | **By CMD status** | | |
| --- | --- | --- | --- | --- |
|  |  | **CMD-free**  (n=11,880) | **CMDs**  (n=1,783) | **P-value** |
| Age, years | 63.4 ± 2.7 | 63.3 ± 2.7 | 63.9 ± 2.8 | <0.001 |
| Sex |  |  |  |  |
| Female | 6,318 (46.2) | 5,787 (48.7) | 531 (29.8) | <0.001 |
| Male | 7,345 (53.8) | 6,093 (51.3) | 1,252 (70.2) |  |
| College/university-educated | 5,740 (42.2) | 5,040 (42.6) | 700 (39.5) | 0.012 |
| White | 12,878 (94.5) | 11,202 (94.6) | 1,676 (94.2) | 0.514 |
| Townsend deprivation index | -2.1 ± 2.6 | -2.2 ± 2.6 | -1.9 ± 2.8 | <0.001 |
| BMI, kg/m^2^ | 26.6 ± 3.9 | 26.4 ± 3.8 | 28.4 ± 4.3 | <0.001 |
| Underweight (<20) | 256 (1.9) | 249 (2.1) | 7 (0.4) | <0.001 |
| Normal (20 – 25) | 4,680 (34.3) | 4,313 (36.3) | 367 (20.6) |  |
| Overweight (25 – 30) | 6,383 (46.8) | 5,522 (46.5) | 861 (48.3) |  |
| Obese (≥30) | 2,328 (17.1) | 1,782 (15.0) | 546 (30.7) |  |
| Smoking |  |  |  |  |
| Never | 7,461 (54.7) | 6,639 (56.0) | 822 (46.21) | <0.001 |
| Previous | 5,557 (40.8) | 4,696 (39.6) | 861 (48.3) |  |
| Current | 616 (4.5) | 518 (4.4) | 98 (5.5) |  |
| Alcohol |  |  |  |  |
| Never | 356 (2.6) | 304 (2.6) | 52 (2.9) | 0.019 |
| Previous | 265 (1.9) | 216 (1.8) | 49 (2.8) |  |
| Current | 13,040 (95.5) | 11,358 (95.6) | 1,6821 (94.3) |  |
| Physical activity |  |  |  |  |
| Low | 1,775 (15.4) | 1,475 (14.7) | 300 (19.8) | <0.001 |
| Moderate | 4,881 (42.3) | 4,259 (42.5) | 622 (41.1) |  |
| High | 4,885 (42.3) | 4,292 (42.8) | 593 (39.1) |  |
| Hypertension | 4,207 (30.8) | 3,219 (27.1) | 988 (55.9) | <0.001 |
| Depression | 4,466 (32.7) | 3,880 (32.7) | 586 (32.9) | 0.863 |
| *APOE* ε4 carrier | 2,963 (25.9) | 2,565 (25.8) | 398 (27.0) | 0.305 |
| CR indicator |  |  |  |  |
| Low | 1,499 (11.0) | 1,279 (10.8) | 220 (12.3) | <0.001 |
| Moderate | 5,147 (37.7) | 4,430 (37.3) | 717 (40.2) |  |
| High | 7,017 (51.4) | 6,171 (51.9) | 846 (47.5) |  |

Data are presented as means ± standard deviations or number (proportion, %).

Missing data: 61 for education level; 41 for race/ethnicity; 6 for Townsend deprivation index; 16 for BMI; 29 for smoking status; 2 for alcohol drinking; 2,122 for physical activity level; 16 for hypertension; 2,227 for APOE ε4 status

Abbreviations: *APOE*=apolipoprotein E; BMI=body mass index; CMDs=cardiometabolic diseases; CR=cognitive reserve

**eTable 7. Comparison of baseline characteristics among participants included vs. not included the neuroimaging subsample.**

| **Characteristics** | **Included in Neuroimaging Subsample**  (n=13,663) | **Not Included in Neuroimaging Subsample**  (n=202,515) | **P-value** |
| --- | --- | --- | --- |
| Age, years | 63.4 ± 2.7 | 64.2 ± 2.9 | <0.001 |
| Sex |  |  | <0.001 |
| Female | 6,318 (46.2) | 107,717 (53.2) |  |
| Male | 7,345 (53.8) | 94,798 (46.8) |  |
| College/university-educated | 5,740 (42.2) | 50,388 (25.2) | <0.001 |
| White | 12,878 (94.5) | 188,414 (93.4) | <0.001 |
| Townsend deprivation index | -2.1 ± 2.6 | -1.5 ± 3.0 | <0.001 |
| BMI, kg/m^2^ | 26.6 ± 3.9 | 27.7 ± 4.6 | <0.001 |
| Underweight (<20) | 256 (1.9) | 3,705 (1.8) | <0.001 |
| Normal (20 – 25) | 4,680 (34.3) | 55,485 (27.6) |  |
| Overweight (25 – 30) | 6,383 (46.8) | 90,740 (45.1) |  |
| Obese (≥30) | 2,328 (17.1) | 51,472 (25.6) |  |
| Smoking |  |  | <0.001 |
| Never | 7,461 (54.7) | 100,011 (49.6) |  |
| Previous | 5,557 (40.8) | 84,263 (41.8) |  |
| Current | 616 (4.5) | 17,210 (8.5) |  |
| Alcohol |  |  | <0.001 |
| Never | 356 (2.6) | 10,003 (4.9) |  |
| Previous | 265 (1.9) | 7,981 (4.0) |  |
| Current | 13,040 (95.5) | 184 (307 (91.1) |  |
| Physical activity |  |  |  |
| Low | 1,775 (15.4) | 27,435 (17.4) | <0.001 |
| Moderate | 4,881 (42.3) | 66,070 (41.8) |  |
| High | 4,885 (42.3) | 64,434 (40.8) |  |
| Hypertension | 4,207 (30.8) | 78,743 (39.0) | <0.001 |
| Depression | 4,466 (32.7) | 31,359 (15.5) | <0.001 |
| *APOE* ε4 carrier | 2,963 (25.9) | 47,236 (28.5) | <0.001 |
| CMDs |  |  | <0.001 |
| CMD-free | 11,880 (87.0) | 160,896 (79.5) |  |
| 1 CMD | 1,620 (11.9) | 34,821 (17.2) |  |
| ≥2 CMDs | 163 (1.2) | 6,798 (3.4) |  |
| CR indicator |  |  | <0.001 |
| Low | 1,499 (11.0) | 57,479 (28.4) |  |
| Moderate | 5,147 (37.7) | 82,124 (40.6) |  |
| High | 7,017 (51.4) | 62,912 (31.1) |  |

Data are presented as means ± standard deviations or number (proportion, %). Missing data: 2,907 for education level; 771 for race/ethnicity; 184 for Townsend deprivation index; 1,129 for BMI; 1,060 for smoking status; 226 for alcohol drinking; 46,698 for physical activity level; 583 for hypertension; 38,937 for APOE ε4 status._._ Abbreviations: *APOE*=apolipoprotein E; BMI=body mass index; CMDs=cardiometabolic diseases; CR=cognitive reserve

**APPENDIX A: Sex Differences**

To explore potential sex differences in the associations of CMDs and CR with dementia and brain structure, the CR latent classes were re-calculated among males and females separately, and sex-specific analyses were conducted. Distribution of CR-related factors in the sex-specific latent classes are illustrated in **eFigure 2.** Results for females are presented in **eTable 8** and results for males are presented in **eTable 9**.

**eFigure 2. Distribution of cognitive reserve (CR)-related factors in the sex-specific low, moderate, and high latent classes.**

**
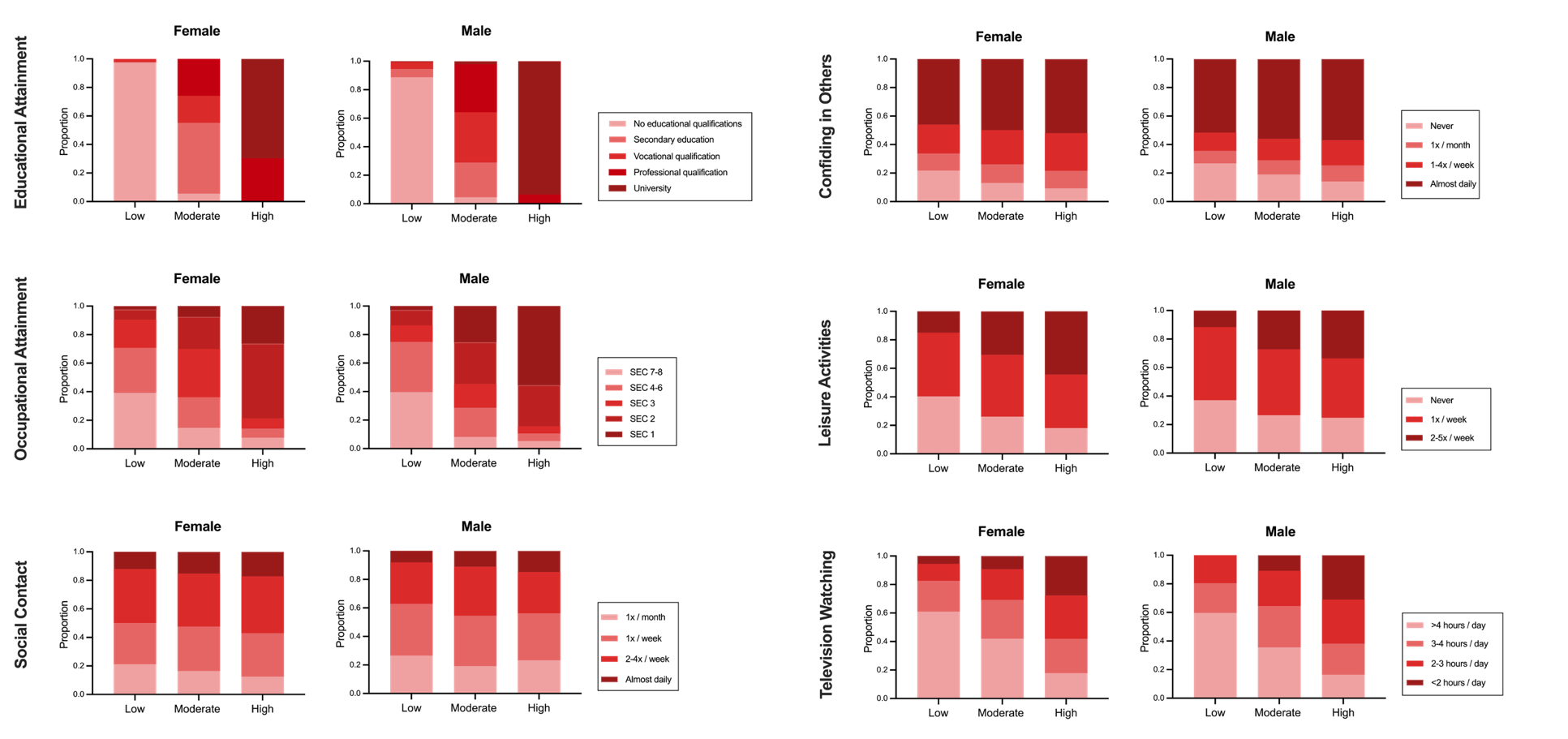
**

Darker colors indicate more favorable and lighter colors indicate less favorable levels of each CR-related factor.

**eTable 8. Summary of main results for females.**

|  | **Dementia** | | **Neuroimaging Measures** | | | | | |
| --- | --- | --- | --- | --- | --- | --- | --- | --- |
|  |  |  | ***n*** | **Gray Matter Volume** | **Hippocampal Volume** | **White Matter Hyperintensity Volume** | **Fractional Anisotropy** | **Mean Diffusivity** |
|  | ***n*** | HR (95% CI)* |  | β (95% CI)** | β (95% CI)** | β (95% CI)** | β (95% CI)** | β (95% CI)** |
| **CMD status** |  |  |  |  |  |  |  |  |
| CMD-free | 97,772 | Reference | 5,787 | Reference | Reference | Reference | Reference | Reference |
| CMDs | 16,263 | **1.81 (1.66, 1.96)** | 531 | **-0.14 (-0.22, -0.07)** | **-0.11 (-0.19, -0.03)** | 0.05 (-0.02, 0.13) | -0.08 (-0.18, 0.01) | **0.10 (0.04, 0.19)** |
| **Cognitive reserve level** |  |  |  |  |  |  |  |  |
| Low CR | 30,511 | Reference | 652 | Reference | Reference | Reference | Reference | Reference |
| Moderate CR | 44,804 | **0.86 (0.79, 0.93)** | 2,352 | **0.16 (0.09, 0.23)** | 0.06 (-0.01, 0.14) | -0.03 (-0.11, 0.04) | -0.01 (-0.10, 0.07) | 0.05 (-0.03, 0.14) |
| High CR | 38,720 | **0.75 (0.69, 0.83)** | 3,314 | **0.29 (0.23, 0.36)** | **0.12 (0.04, 0.19)** | 0.01 (-0.07, 0.08) | 0.02 (-0.06, 0.111) | 0.08 (-0.01, 0.16) |
| **Joint CMD and CR status** |  |  |  |  |  |  |  |  |
| CMD-free, High CR | 73,181 | Reference | 5,202 | Reference | Reference | Reference | Reference | Reference |
| CMD-free, Moderate CR | 24,591 | **1.28 (1.17, 1.39)** | 585 | **-0.21 (-0.28, -0.15)** | -0.07 (-0.14, 0.01) | 0.01 (-0.08, 0.09) | -0.01 (-0.09, 0.08) | -0.07 (-0.16, 0.01) |
| CMD-free, Low CR | 10,343 | **1.90 (1.71, 2.12)** | 464 | **-0.12 (-0.20, -0.04)** | -0.08 (-0.16, 0.01) | 0.04 (-0.04, 0.13) | -0.08 (-0.18, 0.02) | 0.09 (-0.01, 0.19) |
| CMDs, Low CR | 5,920 | **2.12 (1.88, 2.40)** | 67 | **-0.53 (-0.72, -0.34)** | **-0.40 (-0.61, -0.18)** | 0.15 (-0.06, 0.35) | -0.10 (-0.35, 0.15) | 0.08 (-0.17, 0.33) |

*Cox regression models adjusted for age at baseline, race/ethnicity, socioeconomic status, BMI, smoking status, alcohol drinking, physical activity, hypertension, depression, and APOE ε4 carrier status_._

**Linear regression models adjusted for age at baseline, race/ethnicity, socioeconomic status, BMI, smoking status, alcohol drinking, physical activity, hypertension, depression, APOE ε4 carrier status, and MRI-reflated factors (head position, scanner table position, and assessment center).

**eTable 9. Summary of main results for males.**

|  | **Dementia** | | **Neuroimaging Measures** | | | | | |
| --- | --- | --- | --- | --- | --- | --- | --- | --- |
|  |  |  | ***n*** | **Gray Matter Volume** | **Hippocampal Volume** | **White Matter Hyperintensity Volume** | **Fractional Anisotropy** | **Mean Diffusivity** |
|  | ***n*** | HR (95% CI)* |  | β (95% CI)** | β (95% CI)** | β (95% CI)** | β (95% CI)** | β (95% CI)** |
| **CMD status** |  |  |  |  |  |  |  |  |
| CMD-free | 75,004 | Reference | 6,093 | Reference | Reference | Reference | Reference | Reference |
| CMDs | 25,139 | **1.69 (1.57, 1.82)** | 1,252 | **-0.18 (-0.24, -0.13** | **-0.13 (-0.20, -0.07)** | **0.07 (0.01, 0.12)** | -0.04 (-0.11, 0.03) | **0.07 (0.01, 0.14)** |
| **Cognitive reserve level** |  |  |  |  |  |  |  |  |
| Low CR | 27,836 | Reference | 839 | Reference | Reference | Reference | Reference | Reference |
| Moderate CR | 43,513 | **0.83 (0.76, 0.89)** | 2,975 | **0.09 (0.03, 0.16)** | 0.06 (-0.02, 0.13) | 0.01 (-0.06, 0.08) | 0.01 (-0.07, 0.09) | 0.03 (-0.05, 0.11) |
| High CR | 30,794 | **0.73 (0.67, 0.81)** | 3,531 | **0.24 (0.17, 0.30)** | **0.16 (0.08, 0.24)** | -0.01 (-0.08, 0.06) | 0.06 (-0.02, 0.14) | 0.02 (-0.05, 0.10) |
| **Joint CMD and CR status** |  |  |  |  |  |  |  |  |
| CMD-free, High CR | 56,731 | Reference | 5,418 | Reference | Reference | Reference | Reference | Reference |
| CMD-free, Moderate CR | 18,273 | **1.28 (1.17, 1.41)** | 675 | **-0.18 (-0.25, -0.11)** | **-0.10 (-0.18, -0.01)** | 0.01 (-0.06, 0.08) | -0.05 (-0.13, 0.04) | -0.02 (-0.11, 0.06) |
| CMD-free, Low CR | 17,576 | **1.71 (1.56, 1.87)** | 1,088 | **-0.19 (-0.25, -0.13)** | **-0.13 (-0.20, -0.06)** | **0.07 (0.01, 0.13)** | -0.05 (-0.13, 0.02) | 0.07 (-0.01, 0.14) |
| CMDs, Low CR | 9,563 | **2.11 (1.91, 2.34)** | 164 | **-0.32 (-0.46, -0.19)** | **-0.29 (-0.45, -0.13)** | 0.05 (-0.08, 0.19) | -0.04 (-0.21, 0.13) | 0.05 (-0.12, 0.21) |

*Cox regression models adjusted for age at baseline, race/ethnicity, socioeconomic status, BMI, smoking status, alcohol drinking, physical activity, hypertension, depression, and APOE ε4 carrier status_._

**Linear regression models adjusted for age at baseline, race/ethnicity, socioeconomic status, BMI, smoking status, alcohol drinking, physical activity, hypertension, depression, APOE ε4 carrier status, and MRI-reflated factors (head position, scanner table position, and assessment center).

**APPENDIX B: Analyses considering CR-related factors and CMDs separately**

To better understand the contribution of each individual CR-related factor, additional Cox regression models were run to explore the risk of dementia associated with education, occupation, social contact, confiding, leisure activities, and television watching time separately. As with the main analyses, age was used as the time scale and the models were adjusted for age at baseline, sex, race/ethnicity, socioeconomic status, BMI, smoking status, alcohol drinking, physical activity, CMDs, hypertension, depression, and APOE ε4 carrier status. Consistent with the significantly lower risk of dementia observed with moderate CR and high CR (**Table 2**), a similar trend toward lower dementia risk was observed with more favorable levels of education, occupation, confiding, leisure activities, and television watching time, but not social contact (**eTable 10**).

Next, we explored the joint effect of CMD status and CR level on dementia risk in terms of each individual CR-related factor and each individual CMD (type 2 diabetes, heart disease, and stroke). First, six indicator variables were created by combining CMD status (yes vs. no) with each CR-related factor (low vs. moderate vs. high). Second, another three indicator variables were created by combining CR level (high/moderate vs. no) and T2D, HD, or stroke status (yes vs. no). Separate Cox regression models were run using these indicator variables as the exposure, again with age as the time scale and adjusted for age at baseline, sex, race/ethnicity, socioeconomic status, BMI, smoking status, alcohol drinking, physical activity, hypertension, depression, and APOE ε4 carrier status. In the first set of models, exploring each of the six CR-related factors separately, among people with CMDs, having the most compared to the least favorable levels of education reduced dementia risk by 16% (p=0.006), occupation by 26% (p<0.001), confiding by 21% (p<0.001), social contact by 11% (although this difference was not statistically significant; p=0.091), leisure activities by 25% (p<0.001), and television watching by 24% (p=0.001) (**eTable 11**). In the second set of models, exploring T2D, HD, and stroke separately, having moderate/high CR compared to low CR attenuated the risk of dementia among people with T2D by 16% (p=0.004), HD by 14% (p=0.008), and stroke by 3% (although this difference was not statistically significant; p=0.710) (**eTable 12**).

**eTable 10. Hazard ratios (HR) and from Cox regression models for the association between CR-related factors and incident dementia.**

| **CR-related factors** | No. of subjects | **HR of all-cause dementia**  (n=6,600 cases) | **HR of Alzheimer’s disease**  (n=2,866 cases) | **HR of vascular dementia**  (n=1,547 cases) |
| --- | --- | --- | --- | --- |
| **EDUCATION** |  |  |  |  |
| No educational qualifications | 58,891 | Reference | Reference | Reference |
| Certificate of secondary education, O-levels | 34,524 | **0.87 (0.81 – 0.94)** | **0.88 (0.79 – 0.98)** | **0.77 (0.66 – 0.91)** |
| A/AS levels | 39,731 | **0.86 (0.80 – 0.92)** | **0.78 (0.70 – 0.87)** | **0.82 (0.70 – 0.95)** |
| Vocational qualification, national diploma, etc, | 26,307 | **0.89 (0.82 – 0.97)** | 0.93 (0.82 – 1.05) | 0.90 (0.76 – 1.06) |
| College/university | 56,725 | **0.78 (0.73 – 0.84)** | **0.72 (0.65 – 0.80)** | **0.65 (0.56 – 0.76)** |
| *Trend* |  | **0.95 (0.93 – 0.96)** | **0.93 (0.91 – 0.95)** | **0.92 (0.89 – 0.95)** |
| **OCCUPATION** |  |  |  |  |
| Unemployed (SEC-8), routine occupations (SEC-7) | 37,782 | Reference | Reference | Reference |
| Semi-routine occupations (SEC-6), lower supervisory and technical occupations (SEC-5), employers in small organizations (SEC-4) | 42,124 | **0.83 (0.77 – 0.90)** | **0.83 (0.74 – 0.92)** | **0.74 (0.64 – 0.85)** |
| Intermediate occupations (SEC-3) | 35,974 | **0.84 (0.78 – 0.91)** | **0.81 (0.72 – 0.91)** | **0.81 (0.69 – 0.94)** |
| Lower professional and higher technical occupations (SEC-2) | 56,997 | **0.74 (0.69 – 0.80)** | **0.75 (0.67 – 0.83)** | **0.60 (0.52 – 0.70)** |
| Higher professional occupations (SEC-1.2, SEC-1.1) | 43,301 | **0.69 (0.64-0.75)** | **0.73 (0.65 – 0.83)** | **0.59 (0.50 – 0.69)** |
| *Trend* |  | **0.92 (0.90 – 0.93)** | **0.92 (0.90 – 0.95)** | **0.88 (0.84 – 0.91)** |
| **CONFIDING** |  |  |  |  |
| Never | 34,937 | Reference | Reference | Reference |
| Around once per month | 24,596 | 1.01 (0.93 – 1.11) | 1.08 (0.95 – 1.24) | 1.04 (0.87 – 1.24) |
| 1-4 times per week | 42,716 | 0.94 (0.87 – 1.02) | 1.00 (0.88 – 1.12) | 0.94 (0.81 – 1.10) |
| Almost daily | 113,929 | **0.79 (0.74 – 0.85)** | **0.88 (0.79 – 0.97)** | **0.74 (0.65 – 0.84)** |
| *Trend* |  | **0.92 (0.90 – 0.94)** | **0.94 (0.92 – 0.97)** | **0.90 (0.86 – 0.93)** |
| **SOCIAL CONTACT** |  |  |  |  |
| ≤Once per month | 38,807 | Reference | Reference | Reference |
| Around once per week | 70,213 | **0.82 (0.76 – 0.88)** | 0.95 (0.85 – 1.06) | **0.84 (0.72 – 0.97)** |
| 2-4 times per week | 75,856 | **0.93 (0.87 – 0.99)** | 1.06 (0.96 – 1.19) | 0.97 (0.84 – 1.12) |
| Almost daily | 31,302 | **0.90 (0.83 – 0.98)** | 1.02 (0.90 – 1.17) | 0.95 (0.80 – 1.13) |
| *Trend* |  | 0.99 (0.97 – 1.02) | 1.03 (0.99 – 1.07) | 1.01 (0.95 – 1.07) |
| **LEISURE ACTIVITIES** |  |  |  |  |
| No activities | 60,522 | Reference | Reference | Reference |
| 1 activity | 94,915 | **0.90 (0.85 – 0.96)** | **0.90 (0.83 – 0.98)** | 0.94 (0.83 – 1.05) |
| 2-5 activities | 60,741 | **0.76 (0.71 – 0.81)** | **0.79 (0.71 – 0.87)** | **0.68 (0.59 – 0.78)** |
| *Trend* |  | **0.87 (0.85 – 0.90)** | **0.90 (0.85 – 0.94)** | **0.84 (0.78 – 0.90)** |
| **TELEVISION WATCHING** |  |  |  |  |
| ≥4 hours/day | 81,436 | Reference | Reference | Reference |
| 3-3.9 hours/day | 53,211 | **0.86 (0.81 – 0.92)** | **0.88 (0.80 – 0.97)** | 0.91 (0.81 – 1.04) |
| 2-2.9 hours/day | 48,962 | **0.83 (0.78 – 0.89)** | **0.86 (0.78 – 0.95)** | **0.78 (0.67 – 0.89)** |
| <2 hours/day | 32,569 | **0.79 (0.73 – 0.85)** | **0.79 (0.70 – 0.89)** | **0.76 (0.64 – 0.90)** |
| *Trend* |  | **0.92 (0.89 – 0.94)** | **0.92 (0.89 – 0.96)** | **0.90 (0.85 – 0.94)** |

All models were adjusted for age at baseline, sex, race/ethnicity, socioeconomic status, BMI, smoking status, alcohol drinking, physical activity, CMDs, hypertension, depression, and APOE ε4 carrier status_._

**eTable 11. Hazard ratios (HR) and from Cox regression models for the joint effect of CMD status and CR components on risk of incident dementia.**

| **CR-related factors** | **Joint CMD and CR status** | No. of subjects | **HR of dementia**  (n=6,600 cases) |
| --- | --- | --- | --- |
| **Education** | |  |  |
| **Low**: No educational qualifications  **Moderate:** Secondary education, O-levels, A/AS levels, vocational qualification  **High:** College/university | CMD-free, High education | 47,625 | Reference |
|  | CMD-free, Moderate education | 81,263 | **1.14 (1.05 – 1.23)** |
|  | CMD-free, Low education | 43,888 | **1.33 (1.22 – 1.44)** |
|  | CMDs, High education | 9,100 | **1.88 (1.67 – 2.12)** |
|  | CMDs, Moderate education | 19,299 | **1.99 (1.81 – 2.19)** |
|  | CMDs, Low education | 15,003 | **2.23 (2.02 – 2.45)** |
| **Occupational attainment** | |  |  |
| **Low:** Unemployed or routine occupations (SEC-8, SEC-7)  **Moderate:** Semi-routine occupations, lower supervisory and technical occupations, employers in small organizations, intermediate occupations (SEC 3-6)  **High**: Professional and higher technical occupations (SEC 1-2) | CMD-free, High occ. attainment | 82,578 | Reference |
|  | CMD-free, Moderate occ. attainment | 62,445 | **1.18 (1.10 – 1.26)** |
|  | CMD-free, Low occ. attainment | 27,753 | **1.40 (1.29 – 1.52)** |
|  | CMDs, High occ. attainment | 17,720 | **1.78 (1.63 – 1.94)** |
|  | CMDs, Moderate occ. attainment | 15,653 | **2.00 (1.84 – 2.18)** |
|  | CMDs, Low occ. attainment | 10,029 | **2.42 (2.20 – 2.66)** |
| **Confiding** | |  |  |
| **Low:** Never  **Moderate:** Around once per month; 1-4 times per week  **High:** Almost daily | CMD-free, High confiding | 91,695 | Reference |
|  | CMD-free, Moderate confiding | 54,617 | **1.23 (1.15 – 1.31)** |
|  | CMD-free, Low confiding | 26,464 | **1.26 (1.16 – 1.36)** |
|  | CMDs, High confiding | 22,234 | **1.77 (1.64 – 1.91)** |
|  | CMDs, Moderate confiding | 12,695 | **2.13 (1.95 – 2.33)** |
|  | CMDs, Low confiding | 8,473 | **2.23 (2.02 – 2.47)** |
| **Social contact** | |  |  |
| **Low:** Around once per month or less  **Moderate:** Around once per week  **High:** 2-4 times per week; Almost daily | CMD-free, High connection | 85,883 | Reference |
|  | CMD-free, Moderate connection | 56,511 | **0.89 (0.83 – 0.95)** |
|  | CMD-free, Low connection | 30,382 | 1.07 (0.98 – 1.16) |
|  | CMDs, High connection | 21,275 | **1.74 (1.62 – 1.87)** |
|  | CMDs, Moderate connection | 13,702 | **1.56 (1.42 – 1.70)** |
|  | CMDs, Low connection | 8,425 | **1.95 (1.76 – 2.16)** |
| **Leisure activities** | |  |  |
| **Low:** No activities  **Moderate:** 1 activity  **High**: 2-5 activities | CMD-free, High leisure activities | 50,447 | Reference |
|  | CMD-free, Moderate leisure activities | 75,525 | **1.19 (1.11 – 1.28)** |
|  | CMD-free, Low leisure activities | 46,804 | **1.31 (1.21 – 1.42)** |
|  | CMDs, High leisure activities | 10,294 | **1.74 (1.55 – 1.95)** |
|  | CMDs, Moderate leisure activities | 19,390 | **2.08 (1.90 – 2.27)** |
|  | CMDs, Low leisure activities | 13,718 | **2.33 (2.12 – 2.57)** |
| **Television watching** | |  |  |
| **Low:** <2 hours/day  **Moderate:** 2-2.9 or 3-3.9 hours/day  **High:** ≥4 hours/day | CMD-free, Low TV | 27,785 | Reference |
|  | CMD-free, Moderate TV | 83,620 | **1.04 (0.95 – 1.15)** |
|  | CMD-free, High TV | 61,371 | **1.26 (1.15 – 1.38)** |
|  | CMDs, Low TV | 4,784 | **1.64 (1.40 – 1.93)** |
|  | CMDs, Moderate TV | 18,553 | **1.90 (1.71 – 2.12)** |
|  | CMDs, High TV | 20,065 | **2.16 (1.95 – 2.40)** |

All models were adjusted for age at baseline, sex, race/ethnicity, socioeconomic status, BMI, smoking status, alcohol drinking, physical activity, hypertension, depression, and APOE ε4 carrier status_._

**eTable 12. Hazard ratios (HR) from Cox regression models for the joint effect of individual CMDs and CR on risk of incident dementia.**

| **Joint CMD and CR status** | No. of subjects | **HR of dementia**  (n=6,600 cases) |
| --- | --- | --- |
| **Type 2 diabetes** |  |  |
| Diabetes-free, High/Moderate CR | 143,448 | Reference |
| Diabetes-free, Low CR | 51,275 | **1.22 (1.15 – 1.30)** |
| T2D, High/Moderate CR | 13,752 | **1.76 (1.62 – 1.92)** |
| T2D, Low CR | 7,703 | **2.10 (1.90 – 2.32)** |
| **Heart disease** |  |  |
| HD-free, High/Moderate CR | 141,826 | Reference |
| HD-free, Low CR | 50,052 | **1.22 (1.15 – 1.30)** |
| HD, High/Moderate CR | 15,374 | **1.60 (1.48 – 1.74)** |
| HD, Low CR | 8,926 | **1.87 (1.70 – 2.05)** |
| **Stroke** |  |  |
| Stroke-free, High/Moderate CR | 154,106 | Reference |
| Stroke-free, Low CR | 56,994 | **1.23 (1.16 – 1.30)** |
| Stroke, High/Moderate CR | 3,094 | **2.21 (1.92 – 2.54)** |
| Stroke, Low CR | 1,984 | **2.29 (1.96 – 2.69)** |

All models were adjusted for age at baseline, sex, race/ethnicity, socioeconomic status, BMI, smoking status, alcohol drinking, physical activity, hypertension, depression, and APOE ε4 carrier status. Type 2 diabetes, heart disease, and stroke were mutually adjusted for.

**APPENDIX C: Sensitivity Analyses**

**eTable 13. Summary of main results using non-imputed data for covariates.**

|  | **Dementia** | | **Neuroimaging Measures** | | | | | |
| --- | --- | --- | --- | --- | --- | --- | --- | --- |
|  |  |  | ***n*** | **Gray Matter Volume** | **Hippocampal Volume** | **White Matter Hyperintensity Volume** | **Fractional Anisotropy** | **Mean Diffusivity** |
|  | ***n*** | HR (95% CI)* |  | β (95% CI)** | β (95% CI)** | β (95% CI)** | β (95% CI)** | β (95% CI)** |
| **CMD status** |  |  |  |  |  |  |  |  |
| CMD-free | 172,776 | Reference | 11,880 | Reference | Reference | Reference | Reference | Reference |
| CMDs | 43,402 | **1.73 (1.61, 1.86)** | 1,783 | **-0.18 (-0.23, -0.12)** | **-0.11 (-0.16, -0.05)** | **0.07 (0.01, 0.12)** | -0.04 (-0.10, 0.03) | 0.06 (-0.01, 0.12) |
| **Cognitive reserve level** |  |  |  |  |  |  |  |  |
| Low CR | 58,978 | Reference | 1,499 | Reference | Reference | Reference | Reference | Reference |
| Moderate CR | 87,271 | **0.83 (0.77, 0.89)** | 5,147 | **0.13 (0.07, 0.19)** | **0.05 (-0.02, 0.12)** | -0.01 (-0.08, 0.05) | 0.01 (-0.07, 0.08) | 0.05 (-0.03, 0.12) |
| High CR | 69,929 | **0.73 (0.67, 0.80)** | 7,017 | **0.27 (0.21, 0.33)** | **0.13 (0.07, 0.20)** | -0.01 (-0.07, 0.05) | 0.04 (-0.04, 0.11) | 0.05 (-0.02, 0.12) |
| **Joint CMD and CR status** |  |  |  |  |  |  |  |  |
| CMD-free, High CR | 58,738 | Reference | 6,171 | Reference | Reference | Reference | Reference | Reference |
| CMD-free, Moderate CR | 70,493 | **1.13 (1.03, 1.24)** | 4,430 | **-0.13 (-0.17, -0.09)** | **-0.07 (-0.11, -0.02)** | -0.01 (-0.05, 0.03) | -0.04 (-0.09, 0.01) | -0.01 (-0.05, 0.04) |
| CMD-free, Low CR | 43,545 | **1.39 (1.25, 1.53)** | 1,279 | **-0.27 (-0.33, -0.20)** | **-0.11 (-0.18, -0.03)** | 0.01 (-0.06, 0.07) | -0.02 (-0.10, 0.05) | -0.06 (-0.14, 0.01) |
| CMDs, High CR | 11,191 | **1.77 (1.54, 2.02)** | 846 | **-0.17 (-0.24, -0.10)** | **-0.04 (-0.12, 0.04)** | 0.05 (-0.02, 0.13) | -0.01 (-0.10, 0.08) | 0.04 (-0.05, 0.13) |
| CMDs, Moderate CR | 16,778 | **1.97 (1.75, 2.22)** | 717 | **-0.32 (-0.40, -0.24)** | **-0.22 (-0.31, -0.13)** | 0.07 (-0.01, 0.16) | -0.09 (-0.20, 0.01) | 0.06 (-0.04, 0.16) |
| CMDs, Low CR | 15,433 | **2.32 (2.06, 2.62)** | 220 | **-0.45 (-0.59, -0.30)** | **-0.34 (-0.51, -0.18)** | 0.12 (-0.03, 0.27) | -0.14 (-0.34, 0.05) | 0.06 (-0.13, 0.25) |

*Adjusted for age at baseline, sex, race/ethnicity, socioeconomic status, BMI, smoking status, alcohol drinking, physical activity, hypertension, depression, and APOE ε4 carrier status_._

**Adjusted for age at baseline, sex, race/ethnicity, socioeconomic status, BMI, smoking status, alcohol drinking, physical activity, hypertension, depression, APOE ε4 carrier status, and MRI-reflated factors (head position, scanner table position, and assessment center)_._

**eTable 14.** **Hazard ratios (HR) from Cox regression models for the associations of CMD status and CR level with dementia risk, after excluding 610 study participants who developed dementia within the first 5 years of follow-up (n=215,568).**

| **CMD and CR status** | No. of participants | **HR of dementia**  (n=5,990 cases) |
| --- | --- | --- |
| **CMD status** |  |  |
| CMD-free | 172,395 | Reference |
| CMDs | 43,173 | **1.73 (1.63 – 1.83)** |
| **Cognitive reserve level** |  |  |
| Low CR | 58,756 | Reference |
| Moderate CR | 87,046 | **0.85 (0.80 – 0.90)** |
| High CR | 69,766 | **0.74 (0.69 – 0.79)** |
| **Joint CMD and CR status** |  |  |
| CMD-free, High CR | 58,620 | Reference |
| CMD-free, Moderate CR | 70,360 | **1.18 (1.09 – 1.28)** |
| CMD-free, Low CR | 43,415 | **1.39 (1.28 – 1.51)** |
| CMDs, High CR | 11,146 | **1.87 (1.66 – 2.09)** |
| CMDs, Moderate CR | 16,686 | **1.96 (1.77 – 2.16)** |
| CMDs, Low CR | 15,341 | **2.34 (2.13 – 2.58)** |

All models were adjusted for age at baseline, sex, race/ethnicity, socioeconomic status, BMI, smoking status, alcohol drinking, physical activity, hypertension, depression, and APOE ε4 carrier status_._

**eTable 15.** **Sub-distribution hazard ratios (SHR) from Fine & Gray regression models for the associations of CMD status and CR level with dementia risk, accounting for the competing risk of death.**

| **CMD and CR status** | No. of participants | **SHR of dementia**  (n=6,690 cases) |
| --- | --- | --- |
| **CMD status** |  |  |
| CMD-free | 172,776 | Reference |
| CMDs | 43,402 | **1.61 (1.53 – 1.70)** |
| **Cognitive reserve level** |  |  |
| Low CR | 58,978 | Reference |
| Moderate CR | 87,271 | **0.87 (0.82 – 0.92)** |
| High CR | 69,929 | **0.78 (0.73 – 0.84)** |
| **Joint CMD and CR status** |  |  |
| CMD-free, High CR | 58,738 | Reference |
| CMD-free, Moderate CR | 70,493 | **1.13 (1.05 – 1.22)** |
| CMD-free, Low CR | 43,545 | **1.32 (1.22 – 1.43)** |
| CMDs, High CR | 11,191 | **1.72 (1.54 – 1.91)** |
| CMDs, Moderate CR | 16,778 | **1.82 (1.66 – 2.00)** |
| CMDs, Low CR | 15,433 | **2.04 (1.86 – 2.23)** |

All models were adjusted for age at baseline, sex, race/ethnicity, socioeconomic status, BMI, smoking status, alcohol drinking, physical activity, hypertension, depression, and APOE ε4 carrier status_._
